# The assessment of views on ageing: a review of self-report measures and innovative extensions

**DOI:** 10.1007/s10433-020-00556-9

**Published:** 2020-02-24

**Authors:** Verena Klusmann, Nanna Notthoff, Ann-Kristin Beyer, Anne Blawert, Martina Gabrian

**Affiliations:** 1 Department of Psychology, Psychological Assessment & Health Psychology, University of Konstanz, Box 47, 78457 Constance, Germany; 2 Department of Psychology and Human Movement Science, University of Hamburg, Hamburg, Germany; 3 Faculty of Sport Science, Leipzig University, Leipzig, Germany; 4 Charité - Universitätsmedizin Berlin, Berlin, Germany; 5 Department of Social Medicine and Prevention, Institute of Community Medicine, University Medicine Greifswald, Greifswald, Germany; 6 DFG Scientific Network Images of Aging, Constance/Frankfurt, Germany

**Keywords:** Views on ageing, Age stereotypes, Subjective ageing, Self-perceptions of ageing, Assessment, Review

## Abstract

This is a review of existing self-report measures for assessing views on ageing. It provides an overview of instruments, for which basic psychometric properties are available and describes them according to the purposes for which they are suitable. Literature search resulted in the inclusion of 89 instruments which were categorised along eight dimensions. The majority of measures focus on explicit cognitions about people’s own age and ageing or other (older) people. A substantial amount of tools account for the multidimensionality and multidirectionality of views on ageing, i.e. the idea that ageing is accompanied by both gains and losses in several different domains. To some extent, measures reflect that ageing is a long-term process and that views on ageing are malleable, rather than just stable traits. Cluster analysis revealed heterogeneity in instruments regarding the dimensions of *Ecosystem*, *Balance, Stability, Dynamics*, and *Complexity.* It becomes apparent, however, that approaches to measure views on ageing should be extended to more specifically target the implicit level as well as affective, physiological, and behavioural manifestations. Additionally, means for capturing views on ageing on the societal level and tools with a distinct time reference are needed. This is particularly important when one wants to account for the lifelong dynamics of views on ageing.

## Introduction

This review provides an overview of self-report instruments to assess views on ageing (VoA). It categorises them along eight dimensions, thus enabling researchers to choose suitable instruments according to the specific aims and needs of their research questions. It also highlights the gaps in the existing literature and identifies areas in which new or extended measures may be needed.

VoA are defined as a person’s conceptions about older people, old age, and ageing in general as well as conceptions of one’s own age and ageing, that is, subjective ageing (including self-perceptions of ageing and subjective age; Wurm et al. 2017). Hence, prototypical societal conceptions of the competencies, characteristics, and physical conditions of old age and (*other*) older people in general (age stereotypes) must be differentiated from expectations and perceptions of one’s *own* old age or ageing (Miche et al. 2015). These personal experiences are assumed to have a cognitive-evaluative (e.g. Steverink et al. 2001), but also an affective and behavioural component (Diehl et al. 2014). Metastereotypes refer to how we think other people might view older age (Bowen et al. 2011; Staudinger 2015). Against this background, we can distinguish representations of the *status* of being old from those of the *process* of getting old (e.g. Wurm et al. 2017).

It is commonly assumed that the development of VoA starts in early childhood (e.g. Gilbert and Ricketts 2008) and continues throughout life based on embodied stereotypes and personal experiences with older people (Levy 2003, 2009). Resource conflicts, in-group appreciation, and defences against death anxiety (Martens et al. 2005) are assumed to cause the distancing from older people, which is expressed in deviating views on one’s own ageing from those on the ageing of other people. Recently, we reasoned that VoA play a powerful role in shaping development across the entire life span and proposed a lifespan *bio*-*psycho*-*social* approach of views on ageing (Kornadt et al. 2019). Starting in younger ages (e.g. Klusmann et al. 2019) VoA exert their influence on the psychological (i.e. the cognitive and affective level), but also the physiological (e.g. as a reaction to age cues) and the behavioural level (Hess 2006; Levy 2009). In addition, as has recently been summarised by Wurm et al. (2017; Westerhof and Wurm 2018), VoA manifest in well-documented effects on health and well-being. VoA are both products as well as drivers of development running through all bioecological levels (Bronfenbrenner and Crouter 1983), meaning a continuous lifelong interaction with social context on the micro-, meso-, and macro-levels.

Tied to the above, VoA are conceptualised as having an outlasting component (*trait* proportion), but also a variable and malleable component (*state* proportion), as has also been substantiated by recent research (Beyer et al. 2019; Wolff et al. 2014). For instance, a person can have relatively stable age stereotypes, but these could be challenged by stereotype incongruent experiences. It has recently been shown that attitudes toward one’s own ageing undergo substantial changes following critical life events such as impaired health (cf. Kotter-Gruehn 2015; see also Wurm et al. 2019).

Based on the seminal work of Paul Baltes (1987), which describes human ageing as characterised by both developmental gains and losses, VoA are (and hence should be referred to as) *multidirectional*, that is, include both positive and negative aspects of ageing (cf. Wurm et al. 2017). This idea is linked to the issue of complexity, referring to *multidimensionality* (Diehl and Wahl 2010; Diehl et al. 2014) or domain specificity of VoA (Kornadt and Rothermund 2011, 2015). Modern approaches, such as the Awareness of Age-Related Change (AARC) concept, assume that VoA vary on a continuum ranging from sub- or pre-conscious (*implicit*) to conscious (*explicit*; Diehl et al. 2014). Whereas the latter is thought to increase across the life span, subconscious processing decreases (Wurm et al. 2017). A lifespan perspective affords the idea of diachronicity in VoA: VoA point to the *future* in younger people, whereas ageing starts to become part of the *present* towards middle-age, when age-related experiences become part of one’s self-concept. For their evaluations in old age, by contrast, people compare the present to the *past* (cf. Kornadt et al. 2019).

The latter conceptions of VoA seem to be in stark contrast to the classical way of assessing VoA. Measures such as the Attitudes Toward Own Aging (ATOA) subscale of the Philadelphia Geriatric Center Morale Scale (PGCMS; Lawton 1975) as well as the Riegel Scale (Riegel and Riegel 1960) or Palmore’s Facts On Aging Quiz (1977) conceptualise VoA as a unidimensional construct, characterised by unidirectional losses. Through the lack of reference to time and limited measurement invariance of items, most classical approaches to measure VoA face limited applicability to different age groups. ‘Age’ and ‘ageing’ mean different semantic categories for a 5-year-old or a 30-year-old or a 70-year-old, for example. Also, scales such as Attitudes Toward Older People (Tuckman and Lorge 1953), Attitudes Towards Old Age (Eisdorfer and Altrocchi 1961), or Old People (Kogan 1961) emphasise cognitive facets, that is, they largely disregard affective and behavioural aspects and thus seem to hardly map onto the multifaceted and highly individual nature of experiences and perceptions of ageing.

Since the beginning of the assessment of VoA in the 1950s, a wide array of measures has become available to capture them. Most of these had been developed for the purpose of addressing VoA not earlier than in middle adulthood, such as the AgeCog Scales being applied to adults aged 40 years and above (Steverink et al. 2001; Wurm et al. 2007). The present study aimed to review and systematise available measures of VoA to provide answers to the question of how VoA are commonly conceptualised and assessed. This overview reveals which tools of which quality are available for which purpose. Based on the results of this analysis of self-report measures, alternative formats and approaches are considered in terms of their feasibility for supplementation and extension of the established self-report means to assess VoA.

## Methods

### Search strategy

Experts from the DFG Scientific Network Images of Aging were consulted in a focus group to define a preliminary collection of assessment tools or instruments for capturing VoA (*n* = 42) as well as a list of common VoA terms that describe the main concepts (the following 12 in alphabetical order: age anxiety, age identity, age stereotypes, ageing awareness, ageing expectations, ageism, attitudes to ageing, beliefs about ageing, images of ageing, perceptions of ageing, subjective age, and views on ageing). For each of these main concepts, a systematic basic search (topics) in the Web of Science Core Collection electronic database was conducted in combination with an AND operation of ‘assessment OR questionnaire OR measure’. Searches were run from 5 September to 10 October 2018 with no time restriction. In all cases, a comprehensive list of variations (e.g. felt age, desired age, ideal age), synonyms, and MeSH terms (e.g. age/ageing, anxiety/fear, old/older people/adults/elderly/senior), as well as spelling variants and truncations were used. Backwards search for references to potentially relevant measurement tools in the articles was also conducted.

### Inclusion criteria and study selection

Articles, reviews, and book chapters were included as document types (i.e. proceedings and meeting abstracts were excluded). They had to include at least one item or scale on VoA which had not been published elsewhere (in cases where measures were referred to that were not already part of our collection, backwards search was conducted to retrieve the original source and review it for inclusion). Measures in all languages were included if the papers were written in English. The measure had to be publicly available, either free to view through journal subscriptions or provided by the authors on request (*n* = 31, i.e. 7% out of 435 full texts selected through abstract screening were not available). Also, basic psychometric information had to be available, that is, reliability (for multi-item instruments) and at least one validation criterion (e.g. factorial validity, convergent or divergent validity, predictive validity). Inclusion of articles through the literature search was qualified by a second reviewer (V.K. and M.G.). In case of disagreement, records and measures were discussed until consensus was reached.

### Data extraction and analysis

Data extraction and data analysis focused on items, questionnaires, and rating scales developed to capture VoA. A data extraction sheet was developed by the group of authors, and data were extracted from the records of measures accordingly. Besides measure (title or name), authors, and year of publication, the following information was extracted: country of origin, number of citations (Web of Science) as an indicator of frequency of use, number of subscales, names of subscales, number of items (per subscale if applicable), target age group, and purpose (for which the measure was developed). Furthermore, psychometric information on reliability (i.e. Cronbach’s alpha, test–retest, specified other) and validity (i.e. factorial, predictive, divergent, convergent, specified other) was recorded.

A taxonomy to characterise VoA comprising eight dimensions with two to four levels each was developed by the expert panel of the DFG Scientific Network Images of Aging according to conceptualisations of VoA in the literature (see introduction). The first dimension ‘*Ecosystem*’ entails a two-step differentiation. The first step addresses the observer level, that is, the differentiation between individual and society (Whose VoA are addressed, those of the person asked or those of the social context or society as a whole?). In a second step, at the individual level, the target level of observation is further differentiated into self and others (Who are the VoA about, my own age and ageing or other people’s ageing and/or the group of older people in general?). Metastereotypes (see above) are a special case in the latter category.

The second dimension ‘*Dynamics*’ defines whether VoA refer to the status of being old (i.e. VoA refer to a more or less clearly defined situation at a certain point in time or point in life and/or the associations with being a certain age) or the process of getting old (i.e. VoA focus on what changes or remains stable as people grow older). The third dimension ‘*Manifestation*’ distinguishes the four levels on which VoA reflect in a person: VoA at the cognitive level entail thoughts, knowledge, and/or reasoning regarding age, ageing, or older people. On the affective level, there are feelings regarding age, ageing, or older people, and also emotional responses to age cues. On the physiological level bodily responses to ageing occur, in particular to age cues. On the behavioural level actions and conduct associated with own age and ageing (e.g. health behaviour or preparatory behaviour), behaviour directed at other (older) people, or behavioural responses to age cues (e.g. discriminative action) can be differentiated.

The fourth dimension ‘*Stability*’ distinguishes between malleable or stable VoA. On the one hand, changes or fluctuations in VoA reflect differences resulting from distinct states in particular situations (e.g. a phase in life or as a result of acute events and experiences). On the other hand, VoA are assumed to be (relatively) stable, individual characteristics that are part of the personal attitude and value system, that is, traits of personality (cf. Spuling et al. 2019). The fifth dimension ‘*Complexity*’ targets uni- versus multidimensionality of VoA, that is whether these are construed as overarching attitudes on a general level or as specific facets of age and ageing at different levels (e.g. physical, social, psychological) or as divergent VoA in different domains (e.g. work, family, leisure, etc.). The sixth dimension ‘*Balance*’ separates unidirectional from multidirectional VoA conceptions, that is, whether only one valence direction (i.e. age-associated negative aspects/changes or losses) or whether multiple valence directions are considered (i.e. both developmental potentials, gains or neutral phenomena, aside from losses).

The seventh dimension ‘*Awareness*’ differentiates implicit associations of age and ageing and subconscious processes from explicit attitudes. Since implicit VoA cannot be addressed by direct questions that obviously target VoA, more subtle approaches are required, such as indirect assessments (e.g. level of knowledge, consent to prejudice) or behavioural observations (e.g. ignorance, social rejection, age-offensive behaviour). Explicit VoA, by contrast, can be captured by asking straightforward questions about age and ageing without veiling the target (e.g. “older people typically are”, “with my advancing age, I…”). Finally, the eighth dimension *‘Time’* defines whether a reference to a certain time is made. Following a lifespan perspective, VoA can refer to the future, the present, or the past. Future-oriented VoA are directed at anticipated age-related events and/or expectations regarding age and ageing, whereas present-oriented VoA deal with people’s current awareness, their prevailing perceptions and experiences of getting and/or being older. Finally, past-oriented VoA refer to memories or perceptions of things being better or worse and/or different from previously held expectations.

Based on this systematisation, the authors developed a classification scheme with the eight dimensions as main categories and the levels as subcategories, adding a “mixed” subcategory for *Awareness* as well as “mixed (separate subscales)” and “mixed (within one scale)” as subcategories for *Ecosystem*, *Manifestation*, *Dynamics*, *Time*, and *Stability*. For *Ecosystem*, statements on social demands, such as how society should deal with ageing or treat older adults, were coded as ‘societal’, whereas opinions on how older people are treated in society were categorised as metastereotypes and thus coded as ‘other’. For *Stability*, assessments targeting VoA in a certain ‘state’ are often indicated by signal words referring to the current time such as ‘now’, ‘at the moment’, ‘today’, ‘during the last days/weeks/months’. This subcategory usually applies together with the subcategory ‘present’ of *Time*. Of note here is that the three subcategories of future, past, and present are obviously not mutually exclusive: If assessments vary in or mix up time perspectives (e.g. by different items), these are coded in more than one category. For *Awareness*, we coded items that obviously and transparently asked for VoA as explicit, whereas tools that only indirectly (and less apparently for the respondents) assessed VoA, such as Palmore’s Facts on Aging Quiz (1977), for example, were coded as implicit means.

## Results

### Selection of measures

**Fig. 1:**
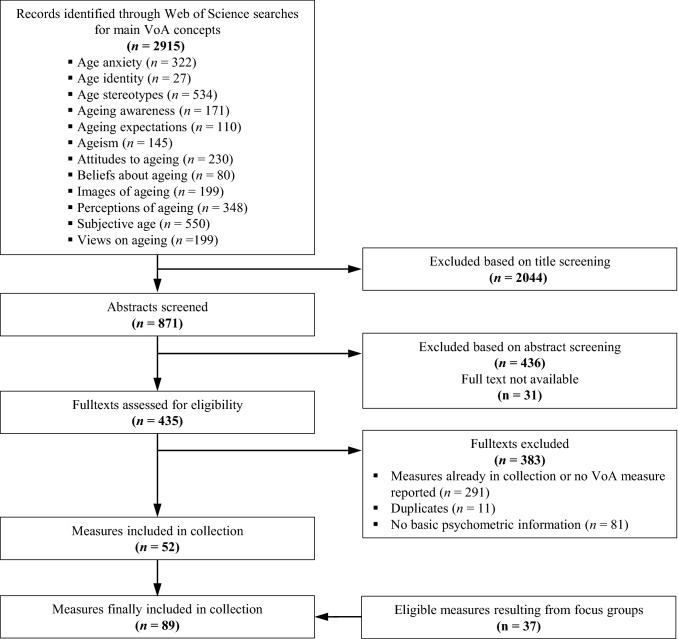
Flowchart for selection of measures based on Web of Science searches and focus groups. Note VoA = views on ageing

Consistent application of the inclusion criteria resulted in the exclusion of five instruments from the preliminary compilation of assessment tools to capture VoA, hence the final collection entailed 37 instruments stemming from the expert consultations. A further 52 instruments were added as a result of the Web of Science searches (see Fig. 1). Thus, the final collection comprised 89 instruments in total (cf. Tables 2, 3, 4, 5).

Characteristics of the measures were recorded using the data extraction sheet and subjected to coding based on the taxonomy as outlined above. Inter-rater agreement was calculated from codings of 50% of the instruments (*n* = 43). Within the eight dimensions, inter-rater agreement ranged from Krippendorff’s *α* = .74 for *Manifestation* to *α* = .90 for *Complexity* (bootstrapping *n *= 1000).

### Global characteristics of measures

Two-thirds (*n* = 59) of the instruments published between 1953 (Attitudes toward older people scale; Tuckman and Lorge 1953) and 2018 (Awareness of Age-Related Change; Brothers et al. 2018) were developed and/or validated in the USA. About two-thirds of the instruments, that is, 32.5% and 31.5%, respectively, either targeted samples of young people under the age of 30 years (mostly students) or included people from adulthood up to old age (i.e. 65 or 85 years and older). The remaining third addressed target samples of 40 + years of age with 21.3% being 60 + year-old samples.

Given the inclusion criteria, basic psychometric criteria were available for all selected measures. Cronbach’s alpha was reported for the vast majority (79%) of instruments (*n* = 70); test–retest reliability was available for 18% of the tools (*n* = 16). More than two-thirds (69%) reported on predictive validity (such as predicting performance, motivation, behaviour, health or well-being); for 47% (*n* = 42), there was information about factorial validity, and reports of divergent and/or convergent validity were available for 34% and 30%, respectively. On average, 15.9 years had passed since publication (median = 11 years) and publications were cited 51.7 times on average (median = 21) with maxima of 2212 Web of Science citations for the broader Stereotype Content Questionnaire (Fiske et al. 2002) used for ratings on diverse target groups and 783 citations for the prominent paper by Lawton (1975) on the Philadelphia Geriatric Center Morale Scale with the well-known Attitudes Toward Own Aging subscale. The number of subscales ranged from zero (i.e. single items or single scale only) to 13.

### Data synthesis on the taxonomy of VoA

#### Dimensionality of VoA

The codings on the eight dimensions defined by the taxonomy are summarised in Table 1. It becomes clear that in currently available self-report assessment tools, VoA are represented in a relatively imbalanced way across most dimensions. Almost 90% of the tools address VoA in an explicit way on its conscious level. Hence, the implicit nature of VoA—being a central attribute of stereotypes—is highly neglected. The vast majority (61%) of the measures addresses VoA on the level of thinking (i.e. cognitive); all measures in the mixed categories of *Manifestation* include cognitive aspects, 68% have a concurrent affective component, and 77% also address behavioural aspects, while only one measure considers physiological matters (i.e. ‘senses’ in the Adults’ Perception of Ages, Montepare 1996a). Two-thirds of the instruments recognise that VoA are multidirectional and, consequentially, also specify age-associated positive aspects and developmental potentials aside from losses. Slightly more than half of the instruments acknowledge the multidimensionality of VoA (56%); however, this means that 44% of the measures give preference to a unidimensional operationalisation of VoA. In terms of *Dynamics*, again two-thirds of the assessments regard VoA as a status quo concept, and only 12% pursue a clear (non-mixed) process perspective. This coincides with 75% of the instruments in which one does not find a time reference. Hence the diachronicity concept is widely disregarded among the instruments. Finally, 88% of the measures regard VoA as a trait.

**Table 1:** Frequencies of VoA dimensions addressed by the selected instruments (*N* = 89)

Level	Dimension	Frequencies	(%)	Adj. (%)
*Awareness*				
1	Implicit	9	10.1	9.7
2	Explicit	79	88.8	89.9
3	Mixed	1	1.1	0.4
*Ecosystem*				
1	Individual: self	18	20.2	23.4
2	Individual: others	38	42.7	37.7
3	Societal	4	4.5	6.5
4	Mixed (separate subscales)	11	12.4	14.2
5	Mixed (within one scale or subscale)	18	20.2	18.2
*Manifestation*				
1	Psychological: cognitive	54	60.7	64.4
2	Psychological: affective	1	1.1	0.3
3	Physiological	0	–	–
4	Behavioural	3	3.4	4.1
5	Mixed (separate subscales)	5	5.6	10.7
6	Mixed (within one scale or subscale)	26	29.2	20.4
*Complexity*				
1	Unidimensional	39	43.8	51.5
2	Multidimensional (separate subscales)	50	56.2	48.5
*Dynamics*				
1	Status	59	66.3	69.9
2	Process	11	12.4	15.9
3	Mixed (separate subscales)	2	2.2	3.6
4	Mixed (within one scale or subscale)	17	19.1	10.7
*Balance*				
1	Unidirectional	29	32.6	35.5
2	Multidirectional	60	67.4	64.5
*Time perspective*				
1	Past	3	3.4	7.2
2	Now	3	3.4	1.9
3	Future	5	5.6	7.6
4	No specific time reference	67	75.3	72.5
5	Mixed (separate subscales)	3	3.4	4.0
6	Mixed (within one scale or subscale)	8	9.0	6.9
*Stability*				
1	State	6	6.7	5.4
2	Trait	78	87.6	84.5
3	Mixed (separate subscales)	0	–	–
4	Mixed (within one scale or subscale)	3	3.4	7.5
5	Unclear	2	2.2	2.6

Adj. (%) = percentage of measures adjusted by mean citation frequency per year; VoA = views on ageing

#### Agents and targets of VoA

Whereas one-third of the instruments assesses a mixture of thinking about others’ age and ageing or old people as a group with thinking about own age and ageing, 43% solely assess responses referring to other (old) people and only 20% are exclusively dedicated to one’s own age and ageing. This individual-level perspective is juxtaposed with at least a quarter of assessments that reflect VoA on the societal level (i.e. *n* = 23, being 26% when counting both pure societal VoA measures and mixed categories).

Contrasting of the groups of instruments rating either only oneself (*n* = 18) to those addressing rating of only others (*n* = 38) for the remaining dimensions underlined that ratings about oneself mean greater introspection. Consequently, despite always entailing a cognitive manifestation, more than 60% of the measures about the ageing self were mixed, entailing also manifestations on the affective (*n* = 6), behavioural (*n* = 6), or physiological level (*n* = 1). For ratings of others, 84% of the measures were purely cognitive, which was a significant difference from self-ratings, *χ*^2^(4) = 19.09, *p* = .001. Also, a process perspective was more likely for ratings referring to the self (44% of process and 44% of status in contrast to 82% of pure status ratings and only 3% of process perspectives reflected in the ratings referring to other people), *χ*^2^(3) = 18.84, *p* < .001. *Complexity* (i.e. dimensionality) and *Balance*, (i.e. gains-losses perspective), however, did not differentiate self-ratings and ratings of others. Consistent with the predominant status concept of VoA, instruments addressing ratings of other people had no time reference at all (100%) and regarded VoA as a trait (97%). Measures referring to self-assessments, however, often referred to the future (28%), the past, or the present (17% each), or mixed these perspectives (22%). In stark contrast to the other-directed measures, only 16% of the self-ratings had no time reference, *χ*^2^(4) = 43.25, *p* < .001. Similarly, different from ratings of other’s age and ageing or old people, only 50% of the ratings referring to one’s own age and ageing regarded VoA as a trait, instead of being a state (28%) or at least mixed state-trait (17%), *χ*^2^(3) = 18.99, *p* < .001.

#### VoA measures across time

Comparing measures published in the last 5 (*n *= 22) or 10 years (*n *= 42) with those of the earlier years did not reveal significant changes regarding the taxonomy profile. There is a tendency of measures having become more process-oriented: 17% of the measures of the last 10 years as opposed to 8.5% of the older instruments. Furthermore, there were tendencies for the measures of the last 5 years to provide slightly more separate subscales to assess manifestations on different (psychological) levels (13.6% vs. 3% for the older measures) and fewer subscales mixing manifestations (31.3% vs. 22.7%), *χ*^2^(4) = 7.81, *p* = .10. Also, of those published in the last 5 years, none continued to mix items with and without time references, compared to 16% of the measures published more than 5 years ago and 21% of the measures older than 10 years, *χ*^2^(2) = 7.33, *p* = .026. Although this trend meant slightly higher proportions of measures with time references in the last 5 years (18% as opposed to only 10% in the older measures), it also coincided with more measures that did not refer to time at all (82% compared to 73% in the instruments of the preceding years).

Further, we explored whether taking into account how much measures are recognised in the scientific community (as indicated by citation frequency) would change the findings regarding the taxonomy profile of measures. Adjusting scores by the mean citation frequency per year did not change results substantially (cf. Table 1). In terms of complexity, the ratio changed slightly, reflecting that unidimensional measures tended to be cited more often than multidimensional measures (the difference was not statistically significant due to the high variability in citation frequencies).

#### Clustering of measures

To identify an overarching structure in how the self-report measures included in this review map on the taxonomy of VoA, a two-step cluster analysis was run using log-likelihood estimation with automatic determination of clusters (max = 15) and model selection via Akaike information criterion (AIC). The resulting four clusters could be differentiated by the way VoA were issued and conceptualised, respectively: Measures in Cluster 1 addressed “VoA as cognitive other-directed multidimensional and multidirectional traits” (*n* = 23 published between 1953 and 2017). Instruments in Cluster 2 covered “VoA as self-directed complex time referenced process” (*n* = 14 published between 1972 and 2018), whereas those in Cluster 3 conceptualised “VoA as mixed multidimensional traits” (*n* = 24 published between 1962 and 2017) and those in Cluster 4 issued “VoA as cognitive unidimensional traits” (*n* = 28 published between 1961 and 2018; see Tables 2, 3, 4, 5).

**Table 2:** Descriptives of instruments in cluster 1 (*n* = 23)

No.	Instrument	Authors	Version/year of publication	Country of origin	No. of citations (WoS)	No. of items	No. of subscales	Citations/year^a^
1	Attitudes toward older people scale	Tuckman and Lorge	1953	USA	247	137	13	3.8
	Tuckman & Lorge questionnaire	Axelrod and Eisdorfer	1961	USA	63	137	13	
	Attitudes towards old people questionnaire	Arnhoff et al.	1964	International	36	67	12	
2	Attitudes toward old age	Eisdorfer and Altrocchi	1961	USA	35	20	4	0.61
3	Aging semantic differential (ASD)	Rosencranz and McNevin	1969	USA	292	32	3	5.96
		Intrieri et al.	1995	USA	30	26	4	
	The German aging semantic differential	Gluth et al.	2010	Germany	27	26	4	
4	Attitudes toward aging	Kilty and Feld	1976	USA	23	45	1	0.55
5	Age stereotypes	Goebel	1984	USA	15	35	6	0.44
6	Stereotypes toward older people scale (STOPS)	Chumbler	1994	USA	n/a	14	4	0.17
7	Perceptions of the elderly	Cheung et al.	1994	China	n/a	18	3	0.13
8	Age group evaluation and description (AGED) inventory	Knox et al.	1995	Canada	28	28	4	1.22
9	Attitudes toward treating elderly patients scale (ATTEPS)	Chumbler	1996	USA	n/a	6	2	0.05
10	Intergenerational exchanges attitudes scale	Stremmel et al.	1996	USA	5	24	5	0.23
11	Stereotype content questionnaire	Fiske et al.	2002	USA	2212	16	4	0.14
12	Image of aging scale	Levy et al.	2004	USA	30	18	2	2.14
13	Beliefs about aging	Fan	2007	Taiwan/USA	n/a	11	5	0.09
14	Perceptions of aging and health behaviour	Huy et al.	2010	Germany	12	31	5	1.5
	German version (reliability reported)	Thiel et al.	2009	Germany				
15	Beliefs and stereotypes about aging	Rust and See	2010	Canada	4	46	3	0.5
16	stereotypical views of younger and older	van Dalen et al.	2010	Netherlands	87	11	2	10.88
17	Evaluative age stereotypes in different life domains	Kornadt and Rothermund	2011	Germany	67	27	8	9.57
18	Ageism attitude scale	Yilmaz and Terzioglu	2011	Turkey	14	23	3	2
19	Personality age stereotypes	Chan et al.	2012	USA (validated in 26 cultures)	39	30	5	6.5
20	Aging stereotypes and exercise scale	Chalabaev et al.	2013	France	10	12	3	2
21	Images of life change (MIDUS)	Kornadt	2016	USA	3	13	4	1.5
22	Successful aging inventory	Lee et al.	2017	USA	1	12	4	1
23	Children’s older adult stereotypes questionnaire (COASQ) based on Hummert 1990	Lineweaver et al.	2017	USA	4	30	2	4

No.	Instrument	Target age group (*N *= sample size)	Purpose	Reliability	Validity
*Descriptives of instruments in cluster 1 (n = 23)*					
1	Attitudes toward older people scale	20–51 years (*N* = 147)	To measure responses to misconceptions and stereotypes about old people in young adults (p. 249)	Retest = 0.36 to 0.62	Predictive
	Tuckman & Lorge questionnaire	Students (age not specified; *N* = 280)	To validate the stimulus-group validity (p. 75)	Retest = 0.36 to 0.62	Predictive; stimulus-group validity via item analysis (agreement, phi-coefficient)
	Attitudes towards old people questionnaire	USA: (*N* = 423), England: (*N* = 245), Sweden: (*N* = 305), Japan (*N* = 184), Greece (*N* = 336), Puerto Rico (*N* = 246)	To study the psychological correlates of attitudes towards old people (p. 43)	Retest = .72 Split half = .85	Predictive
2	Attitudes toward old age	Average age = 21.2 years (*N* = 103)	To compare attitudes toward old persons with attitudes toward average people and the mentally ill (p. 340)	n/a	Predictive
3	Aging Semantic Differential (ASD)	Undergraduate students (age not specified; *N *= 287)	To study the effects of differential social experiences upon subject stereotypes of the ageing individual (p. 55)	n/a	Factor analyses predictive
	Intrieri et al. (1995)	3rd-year medical students (age not specified, *N* = 100)	To evaluate the factor structure of the ASD within this sample (p. 617)	Cronbach’s *α* = .75 to .85	CFA
	The German Aging Semantic Differential	Sample 1: 18–31 years (*N* = 151); sample 2: 68–81 years (*N* = 143)	To evaluate younger adults and to evaluate older adults (p. 147)	Cronbach’s *α* = .59 to .71 (younger)/.81 to .87 (older)	CFA Predictive Divergent Convergent
4	Attitudes toward aging	18–60+ years (*N* = 471), i.e. sample 1: 18–59 years (*N *= 290), sample 2: 60+ years (*N *= 181)	To explore the underlying dimensionality of beliefs about ageing (p. 587)	*h*-squared (communalities)	Factorial
5	Age stereotypes	18–48 years (*N* = 72)	To identify stereotypes held against different age groups (children, adolescents, young, adults, middle-aged adults, old adults, p. 250)	Retest = .75	Predictive
6	Stereotypes toward older people scale (STOPS)	17–60 years (*N* = 292)	To measure college students’ positive and negative stereotypes (un-/favourability) of individuals 65 and older (p. 222)	Cronbach’s *α* = .70 to .77 Retest = .71 to .79	EFA; CFA
7	Perceptions of the elderly	16–78 years (*N* = 240)	To measure perceptions of elderly people (p. 282/283)	Cronbach’s *α* = .59 to .70	CFA
8	Age group evaluation and description (AGED) inventory	17–23 years (*N* = 1400)	To assess how various age groups are viewed (p. 35)	Retest = .57 to .75	PCA
9	Attitudes toward treating elderly patients scale (ATTEPS)	Students (age not specified; *N* = 528)	To develop a scale for a reliable and valid measuring of the attitudes toward treating elderly patients (p. 39)	Cronbach’s *α* = .69	CFA
10	Intergenerational exchanges attitudes scale	20–74 years (*N* = 227)	To assess the psychometric adequacy of a measure of attitudes toward intergenerational exchanges between young children and dependent older adults (p. 317)	Cronbach’s *α* = .60 to .86	PCA
11	Stereotype content questionnaire	Sample 1: average age = 19.9 (*n* = 125), sample 2: average age = 37.9 (*n* = 61), sample 3: average age = 47.7 (*n* = 64), sample 4: average age = 61.1 (*n* = 25), sample 5: average age = 78.4 (*n* = 19; total *N* = 294)	To provide evidence for their stereotype content model (p. 882)	Cluster analysis	Predictive Convergent
12	Image of aging scale	Sample 1: average age = 66.4 years (*N* = 20), sample 2: average age = 69.1 years (*N* = 68)	To measure both positive and negative age stereotypes (p. 208)	Cronbach’s *α* = .82/.84 Retest = .79/.92	Predictive Convergent
13	Beliefs about aging	67–94 years (*N* = 1850)	To capture different dimensions of ageing beliefs (p. 35)	Cronbach’s *α* = .67 to .89	Predictive
14	Perceptions of aging and health behaviour	50–70 years (*N* = 2002; 982 male; average age = 59.9)	To study the relations of perceptions of ageing to healthy diet (p. 381)	Cronbach’s *α* = .83	EFA Predictive Cluster analysis
15	Beliefs and stereotypes about aging	17–43 years (*N* = 140)	To examine undergraduate students’ beliefs about ageing and Alzheimer’s disease (AD) in the cognitive, social, and physical domains (p. 567)	Cronbach’s *α* = .73 to .83 (young target)/. 72 to .81 (old target)/.67 to .83 (Alzheimer target)	Predictive
16	Stereotypical views of younger and older	Employer sample: 21-69 yrs (*N* = 433), employee sample: 20-65 yrs (*N* = 898)	To examine the perception of productivity of older workers by employers and employees (p. 309)	n/a	EFA, CFA predictive
17	Evaluative Age Stereotypes in Different Life Domains	Average age = 35 to 75 (5 birth cohorts; *N* = 700)	To investigate the existence and predictive validity of distinct domain-specific age stereotypes (p. 548)	Cronbach’s *α* = .67 to .86	Factorial Predictive
18	Ageism attitude scale	Average age = 23.2 (*N* = 500)	To develop items for Turkish students (Western culture scales insufficient, p. 261)	Cronbach’s *α* = .67 to .70	PCA
19	Personality age stereotypes	14–90 years (*N* = 3323)	Adaption of the National Character Survey to study age stereotypes (p. 1054)	Cronbach’s *α* = .62 to .77	PCA Accuracy; agreement with self-reported personality data
20	Aging stereotypes and exercise scale	14–89 years (*N *= 714)	To measure the different dimensions of ageing stereotypes in the exercise domain (p. 319)	Cronbach’s *α* = .78 to .84 Retest = .53 to .59	PAF; CFA Predictive Multigroup CFA
21	Images of life change (MIDUS)	50–60 years (*N* = 965)	To test domain specificity in the influence of age stereotypes on personality development (p. 52)	Latent factors, communalities	Efa, Cfa Predictive
22	Successful aging inventory	65+ years, average age = 81.7 (*N* = 550)	To develop a brief multidimensional questionnaire for assessing successful ageing (p. 359)	Cronbach’s *α* = .69 to .75	EFA; Multigroup CFA Predictive Divergent Convergent Measurement invariance
23	Children’s older adult stereotypes questionnaire (COASQ) based on Hummert 1990	6–14 years (*N* = 163)	To assess children’s stereotypes of older adults (p. 303)	Cronbach’s *α* = .76/.83	Predictive Divergent

*WoS* = web of science

^a^Citations/year were calculated as No. of citations per year since publication

**Table 3:** Descriptives of instruments in cluster 2 (*n* = 14)

No.	Instrument	Authors	Version/year of publication	Country of origin	No. of citations (WoS)	No. of items	No. of subscales	Citations/year^a^
24	The ages of me	Kastenbaum et al.	1972	USA	156	6	6	3.39
25	Attitudes toward own aging (as part of the PGCMS)	Lawton	1975	USA	783	5	1	18.21
		Liang and Bollen	1983	USA	210	5	1	
		Miche, Elsässer et al.	2014a	Germany (ILSE)	19	5	1	
26	Reactions to aging questionnaire (RAQ)	Gething	1994	Australia	n/a	27	6	1.33
27	Adults’ perceptions of ages	Montepare	1996a	USA	n/a	16	3	1.55
28	Aging-related cognitions (AgeCog)	Steverink et al.	2001	Germany	95	16	4	5.59
		Wurm et al.	2007	Germany	99		4	
	(Items on Self-knowledge Scale)	Klusmann et al.	2020	Germany	n/a	4	1	
29	Subjective aging perception scale	de Gracia Blanco et al.	2004	Spain	9	12	4	0.64
30	Adult manifest anxiety scale	Lowe and Reynolds	2006	USA	11	44	4	0.92
31	Aging perceptions questionnaire (APQ)	Barker et al.	2007	Ireland	n/a	32	7	4.45
		Sexton et al.	2014	Ireland (Tilda)	20	17	5	
32	Aging concerns	Hostetler	2012	USA	11	4	1	1.83
33	Aging dissatisfaction	Klusmann et al.	2012	Germany	20	6	1	3.33
34	Future self-views	Kornadt and Rothermund	2012	Germany	49	27	8	16.33
35	Retirement stereotypes	Ng et al.	2016	USA	7	14	2	3.5
36	Self-perceptions of aging	Sun and Smith	2017	USA	1	8	1	1
37	Awareness of age-related change	Brothers, Gabrian et al.	2018	Germany (2013); USA (2018)	n/a	50	10	1

No.	Instrument	Target age group (*N* = sample size)	Purpose	Reliability	Validity
*Descriptives of instruments in cluster 2* *(n *= *14)*					
24	The ages of me	20–69 years (*N* = 75)	Personal age (how old a person seems to himself) is proposed (a) as a potential component of total functioning age and (b) as a basis for classification in attempts to create and modify “old behaviour” (p. 197)	n/a	Predictive Divergent Convergent
25	Attitudes toward own aging (PGCMS)	Mean age = 72.6 years (*N *= 828)	To assess the multidimensional inner states of morale (i.e. life satisfaction) of older people (p. 85; p. 89)	Cronbach’s *α* = .81	PCA
	Liang and Bollen (1983)	*N* = 838 (National Senior Citizens Survey as reported by Schooler 1970)		Indicator reliabilities > .19	CFA
	Miche et al. (2014b)	Sample 1: birth cohort 1930–1932, mean age = 43.8 (*N* = 500); sample 2: birth cohort 1950–1952, mean age = 62.5 (*N* = 501)			CFA LGC
26	Reactions to aging questionnaire (RAQ)	20–69 years (*N* = 531)	To measure attitudes towards personal ageing in health professionals (p. 77)	Cronbach’s *α* = .20 to .78	Factorial Divergent Convergent
27	Adults’ perceptions of ages	17–85 years (*N* = 290)	To explore the psychometric properties and predictive value of an alternative measure for indexing age (given that the age adults experience themselves may be a better predictor of their psychological and physical functioning than their actual age, p. 117)	Cronbach’s *α* = .79 to .88	EFA Predictive Divergent Convergent
28	Aging-related cognitions (AgeCog)	40–85 years (*N* = 4034)	Investigating personal ageing experiences and whether and how personal ageing experiences relate to adaptive outcomes, that is, indicators of subjective well-being (p. 365)	Cronbach’s *α* = .77 to .79	Factorial Predictive
	Wurm et al. (2007)	40–85 years (*N* = 1286)		Cronbach’s *α* = .73 to .86	CFA Predictive Divergent Convergent
	Klusmann et al. (2020)	18–92 years (*N* = 1314)	Can VoA predict eating behaviour? (p. 2)	Cronbach’s *α* = .66	Predictive
29	Subjective aging perception scale	61–79 years (*N* = 128)	To assess different dimensions of self-concept and important aspects of well-being (p. 264)	Cronbach’s *α* = .91 to .95	CFA Divergent Convergent
30	Adult manifest anxiety scale	Sample 1: 60+ years (*N* = 226); sample 2: 60–100 years (*N* = 863)	A multidimensional self-report measure designed to assess chronic, manifest anxiety in older adults, aged 60 and older (p. 94)	Cronbach’s *α* = .71 to .88 Retest = .78 to .89 ICC = .72 to .89	Factorial Divergent Convergent
31	Aging perceptions questionnaire (APQ)	65–102 years (*N* = 2033)	Multidimensional and holistic representation of the ageing experience based on Leventhal’s self-regulation model (p. 2)	Cronbach’s *α* = .64 to .89 Test–retest = .61 to .83	CFA Predictive Divergent Convergent
	Sexton et al. (2014)	56–70 years (*N* = 6718)	A concise, multidimensional measure of ageing perceptions (p. 1)	Cronbach’s *α* = .75 to .84	CFA Divergent Convergent
32	Aging concerns	35–75+ years (average age = 51.1, *N *= 136)	To explore the relationship between community involvement and attitudes toward ageing (p. 141)	Cronbach’s *α* = .83	Predictive Divergent
33	Aging dissatisfaction	70–93 years (*N* = 246)	To evaluate the effect of 6-month physical exercise carried out in groups (p. 236)	Cronbach’s *α* = .73	Factorial Predictive
34	Future self-views	30–80 years (*N* = 593)	To study internalisation (age stereotypes into self) and projection (experiences into stereotypes, p. 166)	Cronbach’s *α* = .80	EFA; CFA Divergent Convergent
35	Retirement stereotypes	50–94 years (*N* = 1011)	To examine whether retirement stereotypes are associated with health (p. 69)	French sample = .79/.91	CFA Predictive
36	Self-perceptions of aging	51+ years (average age = 67.3; *N* = 2866)	To examine the association between SPA and healthcare delay over the next 12 months (p. 216)	Cronbach’s *α* = .82	Predictive Latent class analysis
37	Awareness of age-related change	Sample 1: 40–95 years (*N* = 396); sample 2: 40–98 years (*N* = 424)	To capture the inherent multidimensionality and complexity of ageing attitudes (p. 1)	Cronbach’s *α* = .89/.88 (gain/loss)	EFA first sample half; CFA second sample half Divergent Convergent

*WoS* = web of science

^a^Citations/year were calculated as no. of citations per year since publication

**Table 4:** Descriptives of instruments in cluster 3 (*n* = 24)

No.	Instrument	Authors	Version/year of publication	Country of origin	No. of citations (WoS)	No. of items	No. of subscales	Citations/year^a^
38	Attitude toward aging scale	Oberleder	1962	USA	7	25	4	0.13
39	Aging opinion survey	Kafer et al.	1980	USA	54	45	3	1.42
40	Children’s perceptions of aging and elderly	Rich et al.	1983	USA	30	20	1	0.86
41	Fraboni scale of ageism	Fraboni et al.	1990	Canada	110	29	3	3.93
		Rupp et al.	2005	USA	81	25	3	
42	Anxiety about aging scale (AAS)	Lasher and Faulkender	1993	USA	119	20	4	4.76
43	Pigram ageism scales	Braithwaite et al.	1993	Australia	24	52	5	0.96
44	University of California at Los Angeles geriatrics attitudes scale (UCLA-GAS)	Reuben et al.	1998	USA	99	14	1	4.95
		Lee et al.	2005	USA	50	14	4	
45	RAME questionnaire	Parnell et al.	2001	England	n/a	20	1	0.12
46	The Lannacombe enquiry	Parnell et al.	2001	England	n/a	20	1	0.12
47	Expectations regarding aging	Sarkisian et al.	2002	USA	37	38	10	2.31
		Sarkisian et al.	2005	USA	53	12	3	
48	Multidimensional scale for the measurement of agreement with age stereotypes and the salience of age in social interaction	Kruse and Schmitt	2006	Germany	30	24	5	2.5
49	Attitudes to ageing questionnaire (AAQ)	Laidlaw et al.	2007	International	77	24	3	7
		Laidlaw et al.	2018	International	n/a	12	3	
50	Perceptions of elders as…	Kane	2007	USA	18	46	12	1.64
51	Relating to old people evaluation (ROPE)	Cherry and Palmore	2008	USA	35	20	2	3.5
52	Age-based rejection sensitivity questionnaire	Kang and Chasteen	2009	Canada	16	15	1	1.78
53	Contact, anxiety, and young people’s attitudes and behavioural intentions towards the elderly	Bousfield and Hutchinson	2010	UK	55	20	4	6.88
54	Attitudes to old age: meta-perceptions and personal attitudes	Vauclair et al.	2010	UK	n/a	23	8	0.63
55	Negative aging perceptions	Sindi et al.	2012	Canada	13	8	2	2.17
56	Prescriptive intergenerational Tension Ageism Scale	North and Fiske	2013	USA	32	20	3	6.4
57	Ambivalent ageism scale	Cary et al.	2017	Canada	8	13	3	8
58	Explicit age attitudes	Chopik and Giasson	2017	USA	8	7	4	8
59	Allophilia measure	Wagner and Luger	2017	USA	n/a	17	5	2
60	Attitudes toward education for older adults AEOA scale	Kim, Abell et al.	2017a	USA	n/a	43	3	0
61	Attitudes toward older adults	Kim, Lee et al.	2017b	USA	1	1	1	1

No.	Instrument	Target age group (*N* = sample size)	Purpose	Reliability	Validity
*Descriptives of instruments in cluster 3* *(n *= *24)*					
38	Attitude toward aging scale	Average age = 78 years (*N *= 40)	To explore attitudes which differentiated between institutionally adjusted persons and those who presented management-problem behaviour (p. 915/916)	Retest = .75 to .88	Predictive
39	Aging opinion survey	Practitioners sample: average age = 36.8 (*N* = 118); undergraduates sample: average age = 21.9 (*N* = 112)	To separate attitudes toward the current elderly from those toward the ageing process (p. 321)	Cronbach’s *α* = .60 to .78	FA Divergent
40	Children’s perceptions of aging and elderly	Third graders at school with no age specified (*N* = 99)	To investigate the effects of a guidance unit about older persons upon elementary school children (p. 483)	Retest = .73	*t* test comparison of scores of EG and CG after aging lessons
41	Fabroni scale of ageism	16–65 years (*N* = 231)	To include an affective component to supplement the cognitive aspect measured by other instruments (p. 56)	Cronbach’s *α* = .65 to .77/.86 (whole scale)	PCA Divergent Convergent
	Rupp et al. (2005)	Sample A: 17–58 years (*N *= 353); sample B: 17–54 years (*N* = 201)	To mirror theoretical models of ageism that emphasise both cognitive facets and affective facets (p. 335)	Cronbach’s *α* = .70 to .79	CFA Predictive Divergent Convergent
42	Anxiety about aging scale (AAS)	7 cohorts of < 25 years to > 47 years (*N* = 312)	To develop a multidimensional scale to assess ageing anxiety (p. 247)	Cronbach’s *α* = .69 to .78	PCA Predictive Convergent
43	Pigram ageism scales	16–62 years (*N* = 195)	To measure four facets of ageism and to relate them to experimental findings on age discrimination (p. 10/11)	Cronbach’s *α* = .76 to .88	Predictive Convergent
44	University of California at Los Angeles geriatrics attitudes scale (UCLA-GAS)	No age specified (residents, fellow, faculty): sample 1: *N* = 121, sample 2: *N* = 96	To develop and validate a short instrument for measuring general attitudes toward older people and attitudes toward caring for older patients (p. 1425)	Cronbach’s *α* = .76	Predictive Known-groups validity
	Lee et al. (2005)	No age specified; sample 1 (residents): *N* = 177, sample 2 (fellows): *N* = 61	To measure attitudes toward geriatric patients (p. 439)	Cronbach’s *α* = .78 communalities	Factorial Predictive
45	RAME questionnaire	Older adults (age not specified, *N* = 40)	To measure internalised ageism and to clarify its correlation to external ageism (p. 12)	Cronbach’s *α* = .81	Predictive Divergent
46	The Lannacombe enquiry	Sample 1: average age = 79 (*n *= 59, 2-month retest *n* = 45), sample 2: adults (age not specified; *n* = 36), sample 3: students (age not specified; *n *= 30; total *N* = 111)	To measure externalised ageism and to clarify its correlation to internal ageism (p. 12)	Cronbach’s *α* = .81 Retest = .77	Predictive Divergent
47	Expectations regarding aging	65+ year-old patients (*N* = 429)	To rigorously examine the relationship between expectations regarding ageing, health behaviours, service use, and subsequent health (p. 525)	Cronbach’s *α* = .58 to .80	Divergent Convergent
	Sarkisian et al. (2005)	65+ years, mean age = 78 years (*N* = 636; 2001–sample)	See ERA-38	Cronbach’s *α* = .76 to .80	EFA on 1999-data; CFA on 2001-data Divergent
48	Multidimensional scale for the measurement of agreement with age stereotypes and the salience of age in social interaction	40–75 years (*N* = 1275)	To assess age salience and age stereotypes, with particular reference to the contemporary German population (p. 396)	Cronbach’s *α* = .62 to. 69	PCA
49	Attitudes to ageing questionnaire (AAQ)	60–100 years (pilot study: *N *= 1356; field study: *N* = 5566)	To capture the experiences and attitudes of older people in relation to the ageing process (p. 368)	Cronbach’s *α* = .68 to .84 IRT = .74/.81	EFA; CFA
	Laidlaw et al. (2018)	60–97 (99) years (sample 1: *N* = 2487; sample 2: *N *= 2488)	To reduce respondent burden and increase the likelihood of the AAQ-SF being included in research studies (p. 114)	Cronbach’s *α* = .62 to .72	EFA; CFA Predictive
50	Perceptions of elders as…	Age not specified, college students (*N* = 228)	To investigate perceptions of older adults among undergraduate social work and criminal justice students (p. 13)	Cronbach’s *α* = .73 to .91	Predictive Known-groups validity
51	Relating to old people evaluation (ROPE)	18–98 years (*N* = 314)	To measure positive and negative ageist behaviours that people may engage during everyday life (p. 849)	Cronbach’s *α* = .70 Retest = .57 to .72	Predictive Face validity
52	Age-based rejection sensitivity questionnaire	29–102 years (*N *= 2300)	To identify individual differences in vulnerability to ageing stereotypes (p. 312)	Cronbach’s *α* = .91 Retest = .74	PCA Divergent Convergent
53	Contact, anxiety, and young people’s attitudes and behavioural intentions towards the elderly	16–25 years (*N *= 55)	To examine the relationship between intergroup contact and behavioural intentions towards members of the outgroup, to look at age-defined groups, and to examine potential mediators (p. 455)	Cronbach’s *α* = .66 to. 73	Predictive Divergent Convergent
54	Attitudes to old age: meta-perceptions and personal attitudes	Student sample: average age = 17.6 (*n* = 200), 50 + sample: average age = 70.2 (*n* = 200, total *N *= 400)	To develop and establish a reliable limited set of indicators to measure attitudes to age in the UK (p. 1/10)	n/a Interitem correlations: *r*(*ij*)_max_ = .14 to .54	EFA Predictive Divergent Convergent
55	Negative aging perceptions	58–85 years (*N* = 40)	To assess the associations between general and internalised negative ageing stereotypes, depressive symptoms (GDS), subjective and objective memory assessments, and cortisol concentration among older adults (p. 130)	Cronbach’s *α* = .59/.76	Predictive Convergent
56	Prescriptive intergenerational tension ageism scale	16–81 years (total *N* = 2010 across four studies)	To provide a contemporary tool that addresses general equity issues (p. 706)	Cronbach’s *α* = .75 to .87	EFA, CFA Predictive Divergent Convergent
57	Ambivalent ageism scale	Sample 1: 16–32 years (*n* = 397), sample 2: 18–34 years (*n* = 194), sample 3: 18–57 years (*n *= 161), sample 4: 17–36 years (*n* = 32; total *N *= 784)	To add a measure on benevolent ageism, given that there are many scales designed to measure hostile ageism, yet none dedicated to measuring benevolent ageism (p. e27)	Cronbach’s *α* = .91 Retest = .80	EFA Predictive Divergent Convergent
58	Explicit age attitudes	15–94 years (*N* = 704,151)	To examine age differences between implicit and explicit attitudes towards older individuals (p. 169)	Cronbach’s *α* = .57 to .59	Divergent Age differences
59	Allophilia measure	Sample 1: 18–21 years (*N* = 94), sample 2: 55–88 years (*N *= 52)	To assess attitudes toward both younger and older adults (p. 147)	Cronbach’s *α* = .81 to .89 (younger)/.77 to .95 (older people)	Predictive Divergent Convergent
60	Attitudes toward education for older adults AEOA scale	18+ years (students, *N* = 227)	To assess social workers’ attitudes towards education for older adults (p. 342/343)	Cronbach’s *α* = .95	CFA Divergent Convergent Content validity
61	Attitudes toward older adults	Average age = 25.2 (*N* = 72)	To test whether the productive ageing concept may favourably influence students’ perceptions of older adults (p. 149)	Cronbach’s *α* = .83	Predictive

*WoS* = web of science

^a^Citations/year were calculated as No. of citations per year since publication

**Table 5:** Descriptives of instruments in cluster 4 (*n* = 28)

No.	Instrument	Authors	Version/year of publication	Country of origin	No. of citations (WoS)	No. of items	No. of subscales	Citations/year^a^
62	Old people scale	Kogan	1961	USA	356	34	2	6.25
63	Facts on aging quiz	Palmore	1977	USA	278	25	1	6.78
		Palmore	1981	USA	67	25	1	
64	Youth’s attitudes toward the elderly	Sanders et al.	1984	USA	n/a	20	1	2.15
65	Social attitude scale of ageist prejudice	Isaacs and Bearison	1986	USA	52	46	1	1.63
66	Knowledge of aging and the elderly (KAE)	Kline et al.	1990	Canada	16	15	1	0.57
67	Age awareness measure	Montepare	1996b	USA	19	4	1	0.86
68	Ageism survey	Palmore	2001	USA	129	20	1	7.59
69	Peer perceptions of older adults	Pinquart	2002	Germany	33	16	1	2.06
70	Refined Aging Semantic Differential (ASD)	Polizzi	2003	USA	54	24	1	3.6
71	Individual age stereotypes (semantic differential 32 pairs of antonyms)	Rothermund and Brandtstädter	2003	Germany	78	32	1	5.2
72	Stereotyping of the older adult (16-item semantic differential)	Anderson et al.	2005	USA	25	16	1	1.92
73	Age-adapted communication behaviour scale	Anderson et al.	2005	USA	25	16	1	1.92
74	Negative age stereotypes	Kliegel and Zimprich	2005	Switzerland	n/a	6	1	1.62
75	Attitudes toward aging	Lai	2009	Chinese people of China, Hong Kong, Taiwan, Canada, USA	23	5	1	2.56
76	Perceptions of aging measure	Löckenhoff et al.	2009	USA (validated in 26 cultures)	104	8	1	11.56
77	Society’s views of aging	Löckenhoff et al.	2009	USA	104	1	1	11.56
78	Perception of societal age stereotypes	Macia et al.	2009	France/Morocco	10	2	1	1.11
79	Aging attitudes	Mock and Eibach	2011	USA (MIDUS II)	57	2	1	8.14
80	Carolina opinions on care of older adults (COCOA. subscales: ageism and social value of older adults)	Hollar et al.	2011	USA	9	24	5	1.29
81	Age group identification	Weiss and Lang	2012	Germany/Switzerland	111	2	1	18.5
82	Attitudes towards sexuality of older adults scale	Thompson et al.	2014	Canada	8	16	1	2
83	Cultural age stereotype scale	Bernardes et al.	2015	Portugal	1	8	1	0.33
84	Burden views toward older people	Bai et al.	2016	China	23	2	1	11.5
85	Age group identity	Macdonald and Levy	2016	USA	19	5	1	9.5
86	10-pt-feeling-thermometer	Rittenour and Cohen	2016	USA	2	1	1	1
87	Negative general views on aging	Jopp et al.	2017	USA	1	3	1	1
88	Self-perception scale of old age	Mendoza-Nunez et al.	2018	Mexico	1	21	1	1
89	Essentialist beliefs about aging	Weiss	2018	Switzerland	3	4	1	3

No.	Instrument	Target age group (*N* = sample size)	Purpose	Reliability	Validity
*Descriptives of instruments in cluster 4 (n *= *28)*					
62	Old people scale	Three student samples (no age information; total *N* = 482), i.e. sample 1: *N* = 128, sample 2: *N* = 186, sample 3: *N* = 168	To facilitate the study of attitudes toward old people with respect to both norms and individual differences (p. 44)	Spearman-Brown odd–even = .66 to .83	Divergent positive–negative interscale correlations
63	Facts on aging quiz	Over 90 studies from school age to retirement age	To demonstrate misconceptions about ageing and interesting facts about ageing, of which people are unaware (p. ix; in: Palmore 1988)	n/a	n/a convergent face validity
	Palmore (1981)	Students, staff, and faculty	To avoid “practice effects” of FAQ1, when using it to measure training experiences (p. x; in: Palmore 1988)	n/a	n/a convergent face validity
64	Youth’s attitudes toward the elderly	College students (*N* = 157)	To assess attitudes of college students toward six target groups of elderly individuals (p. 59)	Cronbach’s *α* = .90	Predictive
65	Social attitude scale of ageist prejudice	4–8 years (*N* = 144)	To study children’s prejudice against the aged (p. 175)	Cronbach’s *α* = .65 to .70 split half = .87	Predictive divergent convergent photograph validation
66	Knowledge of aging and the elderly (KAE)	19–23 years (*N* = 151)	Alternative to Palmore FAQ (since it is biased by attitude towards ageing, i.e. more items correct if positive attitude, p. 297)	Items correct	Ruling out positive and negative bias (by attitude)
67	Age awareness measure	18–72 years (*N* = 290)	To assess salience of age in self-perceptions (p. 197)	Cronbach’s *α* = .77	Predictive
68	Ageism survey	60–93 years (*N* = 84)	To assess the prevalence of ageism in various societies, prevalent types of ageism, and to identify ageist subgroups of older people (p. 572)	Cronbach’s *α* = .81	PCA
69	Peer perceptions of older adults	60–94 years (*N* = 100)	To compare self-perceptions with perception of age peers (p. 324)	French sample = .68 retest = .85	Predictive
70	Refined Aging Semantic Differential (ASD)	17–22 years (*N* = 350)	To refine the Aging Semantic Differential using a more up-to-date list of adjectives (p. 197)	Cronbach’s *α* = .97 retest = .81 (men)/.79 (women)	PCA divergent
71	Individual age stereotypes (semantic differential 32 pairs of antonyms)	54–85 years (*N* = 690)	To test the validity of contamination, externalisation, and comparison hypothesis, respectively (p. 549/550)	n/a longitudinal profile correlations	Predictive
72	Stereotyping of the older adult (16-item semantic differential)	18–25 years (*N* = 208)	To investigate whether stereotyping processes mediate the effects of various predictors on communication outcomes (p. 268)	Cronbach’s *α* = .91	Predictive
73	Age-adapted communication behaviour scale	18–25 years (*N* = 208)	To test whether certain characteristics and relationships are associated with age-adapted communication behaviours (p. 274)	Cronbach’s *α* = .86	EFA
74	Negative age stereotypes	59–65 years (*N* = 607)	To assess the nature of cognitive complaints in older adults (p. 13)	Cronbach’s *α* = .71	Predictive divergent convergent
75	Attitudes toward aging	55+ years (China: *N* = 500; Hong Kong: *N* = 504; Taiwan: *N* = 500; Canada: *N *= 2272, USA: *N* = 501)	To examine the predictive effects of attitude toward ageing on mental health of ageing Chinese (p. 243)	Cronbach’s *α* = .61	Predictive
76	Perceptions of aging measure	Average age = early 20 years (*N* = 3435)	To capture perceptions of ageing in their complexity with characteristics that could be understood across a wide range of cultural contexts (p. 943)	n/a	Predictive consensus across cultures
77	Society’s views of aging	Average age = early 20 years (*N* = 3435)	To make people rate how positively or negatively they think their culture sees old age (p. 944)	n/a	Predictive
78	Perception of societal age stereotypes	60–92 years (French sample: *N* = 260, Moroccan sample: *N* = 239)	To compare French and Moroccan populations regarding perception of age stereotypes and self-perception of ageing (p. 391)	Cronbach’s *α* = .72 (French sample)/.61 (Moroccan sample)	Predictive
79	Aging attitudes	40+ years (*N* = 1170)	To provide a measure of people’s beliefs about the relative quality of life in older adulthood compared to younger adulthood (p. 981)	n/a	Predictive
80	Carolina opinions on care of older adults (COCOA. Subscales: Ageism and Social Value of Older Adults)	21–34 years (*N* = 110)	To develop a valid and reliable instrument for measuring medical and other health professional students’ attitudes towards older adults and towards a career choice in geriatrics (p. 194)	Cronbach’s *α* = .80	GLS divergent
81	Age group identification	18–85 years (*N* = 544)	To test the hypothesis that older adults will try to avoid the negative consequences of their age group membership by distancing themselves from their age group (p. 153)	Cronbach’s *α* = .72	Predictive
82	Attitudes towards sexuality of older adults scale	18–24 years (*N *= 134)	Because no appropriate explicit measure could be found, we adapted a measure of attitudes towards sexuality in disabled people to measure explicit attitudes towards sex among older adults (p. 262)	Cronbach’s *α* = .86	Predictive divergent
83	Cultural age stereotype scale	65–88 years (*N* = 52)	To investigate the extent to which participants endorsed the cultural stereotypes of older people, i.e. their enduring stereotypical beliefs regarding their own age group (p. 996)	Cronbach’s *α* = .78	EFA
84	Burden views toward older people	60–94 years (*N* = 954)	To examine the effects of burden views toward Chinese older adults on their depressive symptoms (p. 26)	Cronbach’s *α* = .84	Divergent convergent
85	Age group identity	18–75 years (*N* = 800)	To obtain a more complete understanding of the psychosocial factors influencing job satisfaction, commitment, and engagement (p. 174)	Cronbach’s *α* = .91	Predictive
86	10-pt-feeling-thermometer	16–25 years (*N* = 139)	To test the effect of an old age progression simulation on young adults’ reported ageing anxiety and perceptions about older adults as a social group in an experiment (p. 271); the 10-pt feeling thermometer was developed based on Alwin (1997, p. 280)	n/a	Predictive
87	Negative general views on aging	18–99 years (*N* = 151)	To investigate the nature and correlates of young, middle-aged, and older adults’ successful ageing role models (p. 237)	Cronbach’s *α* = .68	Predictive
88	Self-perception scale of old age	60+ years, average age = 66 (*N* = 64)	To evaluate the effect of a healthy ageing program linked to self-perception of old age in Mexican community-dwelling older people (p. 1)	Cronbach’s *α* = .83	Predictive *t*-test on pre-post intervention
89	Essentialist beliefs about aging (EBA)	61–87 years (*N* = 79)	To investigate how individual differences in essentialist beliefs about ageing affect how older adults’ respond to negative age stereotypes. EBA define the process of ageing as fixed and inevitable rather than malleable and modifiable (p. 925)	Cronbach’s *α* = .65 (experiment 1)/.74 (experiment 2)	Predictive

*WoS* = web of science

^a^Citations/year were calculated as No. of citations per year since publication

Specifically, measures in Cluster 1 conceptualised VoA as 100% explicit, cognitive, multidimensional traits without any time reference (see Table 6). The vast majority addressed views on other people’s age and ageing (83%), were multidirectional (i.e. focussed both on gains and losses; 96%), and framed the issue of age or being old as a status (87%). Cluster 2 contained the—mostly explicit (93%)—measures addressing people’s own age and ageing (100%). Subsumed measures comprised both unidimensional and multidimensional approaches (43% and 57%), and fewer unidirectional, solely loss-oriented (36%) than multidirectional (64%) as well as fewer status-oriented (36%) than process-oriented means (57%). Unlike other clusters, items reflected the manifold manifestations of VoA on the individual levels of cognition, affect, and behaviour (71% codings in the mixed categories). All of these instruments referred to diverse time perspectives, that is, past (21%), present (21%), future (36%); and, in some cases, also in a less differentiated mixed manner (21%). Likewise—and in contrast to the other clusters—measures reflected the state and trait proportions of VoA (both 35%), again eventually in a less distinct (21% mixed) or even unclear (7%) way.

**Table 6:** Proportions of VoA dimensions addressed in the four VoA instrument clusters (*N*_total_ = 89)

Level	Dimension	Cluster 1	Cluster 2	Cluster 3	Cluster 4
		*n* = 23	*n* = 14	*n* = 24	*n *= 28
*Awareness*					
1	Implicit	–	7.1	16.7	14.3
2	Explicit	100	92.9	83.3	82.1
3	Mixed	–	–	–	3.6
*Ecosystem*					
1	Individual: self	4.3	100	4.2	7.1
2	Individual: others	82.6	–	12.5	57.1
3	Societal	–	–	–	14.3
4	Mixed (separate subscales)	4.3	–	37.5	3.6
5	Mixed (within one scale or subscale)	8.7	–	45.8	17.9
*Manifestation*					
1	Psychological: cognitive	100	28.6	12.5	85.7
2	Psychological: affective	–	–	–	3.6
3	Physiological	–	–	–	–
4	Behavioural	–	–	4.2	7.1
5	Mixed (separate subscales)	–	7.1	16.7	–
6	Mixed (within one scale or subscale)	–	64.3	66.7	3.6
*Complexity*					
1	Unidimensional	–	42.9	20.8	100
2	Multidimensional (separate subscales)	100	57.1	79.2	–
*Dynamics*					
1	Status	87.0	35.7	54.2	75.0
2	Process	–	57.1	4.2	7.1
3	Mixed (separate subscales)	–	7.1	4.2	–
4	Mixed (within one scale or subscale)	13.0	–	37.5	17.9
*Balance*					
1	Unidirectional	4.3	35.7	45.8	42.9
2	Multidirectional	95.7	64.3	54.2	57.1
*Time perspective*					
1	Past	–	21.4	–	–
2	Now	–	21.4	–	–
3	Future	–	35.7	–	–
4	No specific time reference	100	–	66.7	100
5	Mixed (separate subscales)	–	–	12.5	–
6	Mixed (within one scale or subscale)	–	21.4	20.8	–
*Stability*					
1	State	–	35.7	–	3.6
2	Trait	100	35.7	100	92.9
3	Mixed (separate subscales)	–	–	–	–
4	Mixed (within one scale or subscale)	–	21.4	–	–
5	Unclear	–	7.1	–	3.6

Cluster 1 was labelled “VoA as cognitive other-directed multidimensional & multidirectional traits”; Cluster 2 was labelled “VoA as self-directed time referenced process”; Cluster 3 was labelled “VoA as mixed multidimensional traits”; Cluster 4 was labelled “VoA as cognitive unidimensional traits”. Numbers indicate percentages of instruments collated in the corresponding cluster

The “mixed multidimensional traits” Cluster 3 contained most measures that coevally focused on different levels of the *Ecosystem*. The 83% mixed codings reflected combinations of VoA directed at the self (60%), others (95%), and society (90%). Furthermore, even more mixed manifestations (83%) on the joint cognitive (100%), affective (70%), and behavioural (95%) level appeared. Despite the high amount of explicit measures in the total collection, Cluster 3 had the highest proportion of implicit means (17%), and those with mixed status and process (42%) or different time perspectives (33%). While 79% of the measures were multidimensional, Cluster 3 had the highest proportion of solely loss-oriented unidirectional means (46%).

In stark contrast to the other three clusters, measures in Cluster 4 were exclusively (100%) time unreferenced and unidimensional with a predominant cognitive (86%) focus on other people (57%) and also VoA on the level of society (14% plus 21.5% measures with mixed focus on the different *Ecosystem* levels). Similar to Clusters 1 and 3, measures mostly framed age as a status (75%), of which people have stable assessments (93% trait).

Mean years since publication ranged from 19.8 (median = 14) for Cluster 1, 15.8 (median = 11) for Cluster 3, 14.8 (median = 11.5) for Cluster 2 to 13.8 (median = 9) for Cluster 4. Mean number of citations (mean citations weighted per years since publication in parentheses) ranged from 89.9 (4.51, median = 2.58) for Cluster 2 and 58.4 (4.49, median = 2.10) for Cluster 4 to 40.3 (2.39, median = 1.22) for Cluster 1 and 32.7 (2.96, median = 2.08) for Cluster 3. Hence, by tendency—since these variations were non-significant—Clusters 2 and 4 include somewhat “younger”, well-referenced measures.

Prototypical examples of Cluster 1 are the Attitudes Toward Older People Scale (Tuckman and Lorge 1953) and the Aging Semantic Differential (Rosencranz and McNevin 1969), which congruently capture in an explicit way (i.e. people are aware of what the measure aims at) the multidimensional and multidirectional trait-like status of being old. Both measures refer to (other) older people on the purely cognitive level without any specific time reference (see Table 2).

Examples of Cluster 2 are the established ATOA subscale of the PGCMS (1975) as well as Kastenbaum’s et al. (1972) Subjective Age (SA) measure. Both of these are unidimensional, unidirectional means targeting self-assessment. Whereas ATOA is a classical explicit, cognitive evaluation tool of the ageing process as a mixture of state and trait, SA is an implicit tool whose assessments of the current state are shaped by not only cognitive but also affective representations (in the sense of “feeling” young or old). Both instruments are time referenced: ATOA refers to the past, whereas SA refers to the present status (see Table 3). Prototypical measures collated in Cluster 3 are the Fraboni Scale of Ageism (1990) as well as the Anxiety About Aging Scale (Lasher and Faulkender 1993) for whom the mixture of cognitive but also affective and behavioural manifestation of multidimensional VoA traits is characteristic (see Table 4). They differ, however, in that Fraboni’s scale implicitly targets negative views on other (old) people and loss-oriented societal VoA with no time reference, whereas Lasher and Faulkender’s scale is an explicit one targeting self and others regarding both gains and losses referring to past, present, and future.

Finally, Cluster 4 comprises Kogan’s (1961) Old people Scale, Palmore’s Facts on Aging Quiz, and the more recent Löckenhoff et al. (2009) bisected measure. All of these are unidimensional means addressing purely cognitive, non-time referenced reflections on other (old) people or VoA on the level of society (see Table 5). In contrast to Kogan’s and Löckenhoff’s scale, Palmore’s scale does not capture explicit VoA and works with “wrong” answers (and was therefore also coded as implicit and unidirectional).

## Discussion

The eight-dimensional categorisation of VoA assessment instruments revealed that for some dimensions, existing VoA instruments mostly fall onto one level, whereas for others, different levels of that dimension are already represented within the existing VoA instruments. The latter is the case for *Complexity* and *Balance* as well as for the individual levels of *Ecosystem.* Depending on the purpose and target of the assessment, the pool of self-report measures provides instruments that conceptualise VoA as unidimensional or multidimensional as well as unidirectional or multidirectional and put the focus on either the ageing self and/or other people. This contrasts significantly with the dimensions of *Awareness*, *Manifestation*, and *Time Perspective*, and—at least to a certain degree—*Dynamics* and *Stability*.

These patterns of central foci of assessment are mirrored in the clusters which map VoA instruments based on co-occurring dimensions. Whilst there is divergence concerning *Ecosystem*, *Balance* (esp. Cluster 1)*, Stability, Dynamics* (esp. Cluster 2), and *Complexity* (esp. Cluster 4), there is little to no differentiation concerning *Awareness* (all explicit), *Manifestation* (prevailingly cognitive), and *Time Reference* (three quarters of the instruments do not refer to time). This hints at where the current assessment of VoA should be extended.

### Buzz and balance for self-report assessments of VoA

Overall, the collection of instruments works perfectly well for the assessment of individual VoA on the explicit and cognitive level. There are differentiated measures to capture self-views as well as one’s way of thinking about other people’s age and ageing or old people as a group (age stereotypes, in particular). There are tools to capture rough and global VoA or very well elaborated and validated ones that allow studying domain-specific, multidimensional VoA. Two-thirds of the instruments reflect the idea that age and ageing infer both gains and losses; a minority of the instruments includes just one valence direction. There are some decent options for mapping changes in VoA, such as lifelong dynamics (i.e. the process of ageing) or fluctuations (i.e. states as opposed to stable trait-like VoA). The finding, however, that the vast majority of the measures regard VoA as a trait is particularly remarkable against the background of current attempts to find effective ways of intervening to dissolve traditional age stereotypes and allow for more differentiated VoA in terms of productive development (e.g. Beyer et al. 2019). These efforts are based on the idea that VoA are indeed malleable and thus, at least in part, reflect a state.

What the collection seems to miss are means to capture the implicit aspects of VoA, their manifestation in affect, physiology, and behaviour as well as their societal representation. Also, regarding VoA as a lifelong phenomenon (see Kornadt et al. 2019) cannot forego consideration of its diachronicity: Depending on life phase VoA are differentially tied to one’s past, present, or future. Yet the vast majority of self-report measures neglect to include time references. Instruments exclusively targeting one’s own age and ageing were somewhat more differentiated in terms of manifestation on distinct levels, considering the malleability of VoA (VoA also as states), referring to ageing as a process, and regarding time references. This finding is limited, however, by the fact that the instruments purely targeting one’s own age and ageing (*n* = 18, i.e. 32%) were outnumbered by those on others’ age and ageing (*n* = 38, i.e. 68%). Similarly, the most recently published means tended to be less mixed (i.e. using separate subscales to differentiate diverse manifestations, including references to time) and slightly more process-oriented. Measures with such a profile were among the highly cited ones in Cluster 2. Meanwhile, none of the measures published over the last 5 years were implicit.

The inclusion criteria chosen for the selection of instruments for our systematisation may have contributed to certain aspects being underrepresented: We exclusively considered self-report questionnaire measures. This format might lead to approaching phenomena on the explicit and cognitive level and focus on the individual—both as perceiver and as target. In contrast, linguistic and literature content analyses are much more common in sociological research traditions (Ng et al. 2015). Moreover, other forms of psychological assessment typically used in basic research, such as reaction time tasks like implicit association tests (Greenwald et al. 1998) or subliminal priming (Elgendi et al. 2018) are specially thought to address phenomena on implicit and affective levels. Diary entries for the natural occurrence of ageing experiences (Miche et al. 2014b), or ecological momentary assessments (Kotter-Gruehn et al. 2015) allow capturing intra-individual variability and systematically operationalising time references. A review of methodological alternatives to pure self-report and classic questionnaire measures could indicate to what extent these may provide useful extensions for filling the gaps found.

### Thinking outside the box for innovative extensions

Most of the alternative measures that are addressed in this section do not fall under the labels commonly used to describe ‘views on ageing’ as outlined above. Hence, these suggestions are not meant as definitive recommendations. The actual suitability of these approaches in supplementing the self-report instruments systematised in the present study would be subject to future research.

What became clear is that the implicit part of VoA as well as their manifestations on the affective and behavioural level appear underrepresented in self-report measures. Additionally, means of time reference and possibly those that allow depicting the lifelong ageing processes as well as fluctuations in people’s VoA over time would be worth a closer look. The classical tool for studying phenomena on the subconscious, that is, implicit and affective level, is the Implicit Association Test (IAT; Greenwald et al. 1998), a very popular method in experimental research. Frequently combined with psychophysiological measures, which allow capturing pre- and sub-conscious processes as well, the IAT compares reaction times as responses to pairings of specific concepts (e.g. old vs. young) with certain attributes (e.g. positive vs. negative; Hummert et al. 2002). A version for children has also been developed (Babcock et al. 2016). Another reaction time-based method for assessing VoA, specifically age stereotypes, is the so-called contradiction paradigm. Here, participants’ reading times for sentences with content that is either consistent or inconsistent with negative age stereotypes are compared (Lassonde et al. 2012). In terms of the VoA dimensions presented in the present review, these tests target the implicit level on the *Awareness* dimension as well as affective and—depending on the concrete operationalisation—physiological levels of *Manifestation*. The same is true for experiments using priming, which additionally often target the behavioural consequences of VoA. As such, experiments examine people’s performance in cognitive testing and daily functioning (e.g. driving) after being exposed to negative age stereotypes (e.g. Chapman et al. 2014; Hagood and Gruenewald 2018; Mazerolle et al. 2012). Another approach involves manipulating people’s subjective age and examining the influence of the manipulation on various outcomes (Eibach et al. 2010).

Diverse stimuli related to age, ageing, and older people are used in related paradigms. Photographs of younger and older faces are used, for instance, to study relationships to ratings of attractiveness or intelligence or associations between negative/positive expressed emotions and age ratings (Kotter-Gruehn and Hess 2012; Palumbo et al. 2017). Voice samples (Montepare et al. 2014) and vignettes (Schroyen et al. 2016) are also popular stimuli to study age stereotypes on an implicit level, while—depending on design—also allowing observation of affective, physiological, and behavioural manifestations.

Ageing simulation takes a somewhat different approach to address ageing in a holistic way on different levels of *Awareness* and *Manifestation*. Younger and middle-aged adults are made to experience “ageing” or their “older selves”. This includes simulated ageing exercises, in which participants wear ageing suits or other apparel to let them experience sensory and physical changes while studying their affect, behaviour, and cognitions (Green and Dorr 2016). Similarly, virtual reality experiments allow participants to see and interact with an aged version of their self and measure their reactions and behaviours, that is, how close they feel to their future aged self or how much money they allocate to retirement funds, for example (Hershfield et al. 2011). These approaches also entail a *Time Reference*, in that they make participants experience aspects of their possible future.

A method that also relies on self-report, but not on the classical questionnaire format, are diary studies, in which participants report, for example, on experiences related to age and ageing on a daily basis (Miche et al. 2014b). In the age of digitalisation, sophisticated ecological momentary assessments (Kotter-Gruehn et al. 2015) provide an excellent tool to explicitly separate state-trait proportions of VoA (the *Stability* dimension) as well as—if set up as longitudinal studies—to study lifespan ageing processes in combination with changes in VoA over time (the *Dynamics* dimension). These tools also allow for an elaborated study of diachronicity phenomena of VoA across the life span (the *Time Reference* dimension), being highly parsimonious and user friendly at the same time.

Last but not least, taking a more sociological perspective on VoA both in terms of theory and assessment might help supplement overly individualistic assessments of VoA. There are studies relying on interview techniques (Horton et al. 2008), content analyses of media, such as site descriptions on social media like Facebook (Levy et al. 2014), analyses of linguistic databases (Ng et al. 2015), identification of adjectives used to describe “typical” older people (Chen and King 2002; Wehr and Buchwald 2007), or generation of words describing older people’s activities (Wurtele 2009), to name just a few. Like self-report measures, these approaches are language-based but instead mostly address a combination of implicit and explicit VoA on both individual and societal levels and allow studying societal trends and dynamics in VoA. Recently, photographs about ageing taken by different age groups were used as an indicator of VoA in everyday life (Klusmann 2020).

As mentioned above, this list of alternative approaches is not exhaustive. A fertile extension of self-report measures of VoA might also involve methods from disciplines even further remote than those described. What becomes clear, however, is that overcoming disciplinary boundaries seems promising for the development of innovative extensions to assess VoA dimensions in a more complete way.

## Limitations and outlook

One of the major strengths of this review is that its taxonomy and categorisation is theory-based and goes far beyond other recent efforts to collect and report on VoA measures, both in scope and in content (e.g. Ayalon et al. 2019; Faudzi et al. 2019). Furthermore, it relies on an extensive search that combines multiple approaches and sources, i.e. focus groups with experts as well as a systematic literature search. This comprehensive search strategy resulted in a collection of 89 instruments that allows for a valid and high-quality overview of how VoA have been measured over decades.

Although Web of Science is a database that covers a wide range of disciplines, a literature search in several different databases could have helped to reach even higher certainty that truly all existing VoA measures have been considered for inclusion in the review. Furthermore, whereas the search terms for VoA were quite comprehensive, the use of additional keywords representing ‘instrument’ (e.g. screening, inventory, tool, profile) might have led to the identification of a few more VoA measures. Adding these terms to two of the search’s subsets on main concepts as outlined in the methods section, however, did not reveal any further hits.

In considering the strengths and limitations of this review, one also has to keep in mind that strict inclusion and exclusion criteria also always mean trade-off decisions. The criterion to only include tools with basic psychometric information available meant that 81 instruments were excluded; these were mostly developed ad hoc to study ageing attitudes as endpoints of research. Including these in our classification may have changed the answer to our question of how VoA are measured. Nevertheless, in our view, the systematisation and categorisation reported here along with considerations about innovative extensions and combinations of measures with tools that typically do not run under the label of VoA allow for a sound overview of available instruments to assess VoA, maybe even across the whole life span.

Given that roughly two-thirds of the instruments in our collection originate in the USA, it is likely that the included instruments reflect mostly Western, and particularly North American ideas with regards to VoA (Löckenhoff et al. 2009; Voss et al. 2018). Furthermore, two-thirds of the instruments included here have been validated with adult and older adult samples only. As outlined in the introduction, not all of these measures seem appropriate for straightforward use with other age groups and would need adaptations because ‘age’ and ‘ageing’ have different meanings for a 70-year-old than a 30-year-old or a child. Hence, there is a need for validated measures that can be used across the life span.

## Conclusion

This review provides an overview of self-report instruments to assess VoA and categorises them such that researchers and practitioners can choose the appropriate instrument for their project. We found that VoA are already being measured on a wide range of dimensions. That is, we have well-validated tools to assess VoA both in terms of either thinking about oneself as ageing or being old as well as evaluations of other people being old or getting old. There are tools to study VoA in a parsimonious unidimensional way in the form of global ratings, but also elaborated measures that allow considering the multidimensional nature of both ageing and VoA by differentiating specific domains. Most instruments reflect that ageing encompasses both gains and losses and is not a unidirectional phenomenon. There are also means to regard ageing as a process and some tools that refrain from regarding VoA as stable traits, but provide options to assess changes in VoA. However, we saw that the issue of time frames in which VoA develop and occur is underdeveloped—a phenomenon that seems tied to the observation that only recently have researchers started to regard VoA from a lifespan perspective. We have learned that widening the focus to approaches that do not typically come under the label of VoA might enable us to assess a more complete range of dimensions than the current self-report measures of VoA do. Hence, the predominant focus on explicit individual VoA manifested on the cognitive level might be extended to include also the implicit proportions of VoA along with their affective, behavioural, and maybe also physiological manifestations. Societal VoA might become a stronger issue and—following the idea of a lifelong role of VoA—time references across the life span could be emphasised, along with taking a process perspective and considering situation-specific variations of VoA.
